# Identity leadership and cohesion in elite sport: The mediating role of intra-team communication

**DOI:** 10.1016/j.heliyon.2023.e17853

**Published:** 2023-07-03

**Authors:** Gaute S. Schei, Rune Høigaard, Martin K. Erikstad, Andreas Ivarsson, Tommy Haugen

**Affiliations:** a Department of Sports Science and Physical Education, University of Agder, Kristiansand, Norway; b School of Health and Welfare, Halmstad University, Sweden

**Keywords:** Leadership, Social identity, Performance, Communication, Mediation

## Abstract

One perspective on leadership that has recently gained increased attention in sport is identity leadership; however, research on elite sport teams is still in its infancy. Thus, the main purpose of this research is to investigate identity leadership in elite team sports in relation to task cohesion, and subsequently to explore the mediating role of the intra-team communication dimensions of acceptance and distinctiveness. A cross-sectional design was employed and 441 elite athletes from division 1 and division 2 in handball and ice hockey participated in the present study. Structural equation modeling was used to assess relationships between identity leadership and task cohesion, and the mediating role of acceptance and distinctiveness. Results revealed that identity leadership positively predicted task cohesion, and that this relationship was mediated by intra-team communication acceptance. In conclusion, findings in the present study expand our understanding of performance-related benefits of identity leadership in elite sport.

## 1 Introduction

Research into elite team sports has highlighted the benefits of adaptive group dynamics, including effective communication, collective efficacy, and cohesiveness [[1], [2], [3]]. According to the latter, cohesion is one of the most investigated group dynamic constructs and in sport it has commonly been defined as “a dynamic process that is reflected in the tendency for a group to stick together and remain united in the pursuit of its instrumental objectives and/or for the satisfaction of members affective needs” [4, p.213]. Cohesion has been associated with a variety of positive group and individual outcomes, including reduced social loafing [5], needs satisfaction [6], and athlete satisfaction [7]. Of particular relevance for elite sports contexts, cohesion has also been linked to increased performance [8,9]. While both task and social cohesion are seen as highly important for sports teams, studies have found that coach leadership behaviors are more strongly associated with task cohesion than with social cohesion [10,11]. Notably, task cohesion can be considered particularly relevant for elite sports groups, given their focus on performance-related objectives [12] and the positive relationship between task cohesion and collective efficacy in professional team sports [1,13].

While the coach is generally highlighted as highly influential in facilitating group dynamics in sports [14], Miller et al. [15] have noted that this is particularly relevant within competitive sport, where the coach is the leader who represents the group and inspires athletes to unite and mobilize their efforts. While the term leadership generally describes the process whereby an individual influences a group of individuals to achieve a common goal [16], coaches' leadership in competitive sport groups has been investigated through different leadership theories, such as transformational leadership [17] and servant leadership [18]. Recently, there has been increased interest in social identity leadership in sport, and how identity leadership may create successful sports groups [19]. Despite some conceptual overlap between different leadership theories, findings from research focusing on other types of leadership may not be transferrable to identity leadership. Given the theoretical reasoning that identity leadership enhance the power of the collective through a shared identity among team members, it differs from other leadership theories that often emphasizes on the attributes of leaders as individuals [20]. More specifically, as identity leadership stimulates followers to embrace a shared social identity, transformational leadership emphasizes on leaders’ ability to inspire followers to reach their potential as individuals [21].

Social identity leadership is grounded in a social identity approach [22] which recognizes individuals' personal identity and the various social identities they share with others (e.g., team members). According to the theory, experiencing a shared social identity will create a sense of belonging to the group, and will influence athletes' perceptions and behaviors. The social identity approach to leadership proposes that the following four principles lay the foundation for identity leadership: a) identity prototypicality (the leader represents the identity that defines the group they lead), b) identity advancement (the leader promotes the group's interests – “doing it for us”), c) identity entrepreneurship (creating a sense of belonging), and d) identity impresarioship (the leader develops and executes events, activities, and structures that foster the group's sense of shared social identity) [23,24]. Thus, the theory proposes that successful leadership is a process of social influence, where a coach's engagement in the four principles will strengthen group members' social identification with the team. In accordance with the theoretical predictions, there is evidence that perceptions of identity leadership by the coach are positively associated with team identification [25]. Furthermore, studies have found positive associations between social identity and adaptive outcomes, such as collective efficacy [26], commitment [27] and cohesion [28].

A growing body of research on identity leadership in sport contexts has been conducted over the past decade. For instance, in an early study on identity leadership, Slater et al. [29] performed a thematic analysis of media data focusing on six leaders from the 2012 Olympic Games. They found that the more successful leaders communicated in accordance with the principles of identity leadership, for instance, by promoting a collective language. Furthermore, Miller et al. [15] conducted two studies with amateur and professional athletes. In their first (cross-sectional) study, positive relationships between identity leadership and self-efficacy, control, approach goals, and social support were identified. They also found that these relationships were mediated by relational and group identification. In their second study, perceptions of identity leadership at the beginning of the season were related to athletes’ self-efficacy at the end of the season, mediated by relational identification. Similarly, Brunauer et al. [30] examined relations between identity leadership and social identification over the course of a season using social network analysis with sports teams, finding a mutually reinforcing bidirectional link between identity leadership and social identification. In addition, Herbison et al. [21] highlighted that coaches engage in identity behaviors in a variety of social environments before, during and after competition. Their findings indicate that youth coaches use specific behaviors to influence the social environment of their team, in line with dimensions of identity leadership. However, it should be noted that results also suggest that coaches used principles of identity leadership in ways that can undermine positive athlete experiences in youth sport.

Although research on the link between identity leadership and cohesion is still in an initial phase, there is a substantial theoretical link between identity leadership and cohesion. For instance, according to the definition provided by Steffens et al. [31, p.1004], leaders’ identity entrepreneurship will make athletes “feel that they are part of the same group and increasing cohesion and inclusiveness within the group”. Furthermore, Worley et al. [28] found that social identity mediated the relationship between peer servant leadership and cohesion. More specifically regarding the relationship between identity leadership and task cohesion, Steffens et al. [31; Study 4] in a study among sporting teams in Belgium found task cohesion to be predicted by both identity impresarioship and identity entrepreneurship, while identity prototypicality and identity advancement did not predict task cohesion.

To advance the understanding of identity leadership in sport and exercise, Stevens et al. [19] pointed out in their review that more research from the elite sports population is warranted, as studies on identity leadership have been restricted to observational and anecdotal evidence [29]. Furthermore, Stevens et al. [19, p.8] noted that “it is important to test and establish the mechanisms through which identity leadership affects key outcomes”, thus highlighting the need to investigate potential mediators. Moreover, Carron and Spink [32] highlighted in their model for cohesion in sports teams that leadership factors (e.g., identity leadership) influence team cohesion through adaptive group processes. Communication within the team is highlighted as one such group process that is influenced by leadership factors, that in turn will influence cohesion [33]. In support of this model, intra-team communication was found to mediate the relationship between transformational leadership and task cohesion [34]. These findings are also in line with those of Hardy et al. [35], who offered support for a mediating role of intra-team communication on the leadership–task cohesion relationship among athlete leaders in sports teams. To aid in the systematic process of studying communication, Sullivan and Feltz [36] present different aspects of effective communication in team sports. Of particular interest in the current study is the dimensions of acceptance (e.g., messages that support team members) and distinctiveness (e.g., messages that promote a shared and inclusive identity). It is reasonable to believe that in an elite team sport context, acceptance communication contributes to enhanced perceptions of task aspects of cohesion [3] and higher levels of trust [37]. Moreover, it is reasonable to assume that distinctiveness communication increase togetherness through valuable interactions among team members that promote the ingroup [38]. Taken together, acceptance and distinctiveness intrateam communication may strengthen the team as a collective performance unit and increase task cohesion, and subsequently contribute to team performance [39].

Thus, the purpose of the present study was to investigate the relationship between identity leadership and task cohesion in elite team sport, and to further study the potential mediating role of intra-team communication. While the relationship between identity leadership and cohesion is theoretically well founded [31], to our knowledge, the relationship has not been investigated empirically among elite team athletes. In line with studies finding positive relationships between leadership behaviors focusing on unifying the group (i.e., transformational leadership) and cohesion [17,34], we expected a positive relationship between identity leadership and task cohesion. Furthermore, we hypothesized that identity leadership influences the communication within the team; previous studies have found intra-team communication is related to cohesion [38,40]. Additionally, intra-team communication has been found to serve as a mediator between leadership styles and task cohesion [34]. Thus, we expected intra-team communication to mediate the relationship between identity leadership and task cohesion.

## 2 Materials and methods

Ethical approval for the current study was given by the Norwegian Social Sciences Data Service and the Faculty Ethical Board at the first author's university. The sample in this study has been used in one other article [41], but that article referred to a different research question.

### 2.1 Participants

A total of 441 elite athletes from handball (*n* = 295) and ice hockey (*n* = 146) in Norway participated in the study. Mean age of the participants was 21.99 years with a standard deviation of 4.29 and range of 16–39 years. A total of 28 teams participated: 19 handball teams (153 males, 142 females) and nine ice hockey teams (108 males, 38 females). Overall, 14 teams (218 players) played in the highest senior division, and 14 teams (226 players) played in the second highest division. The number of players per team ranged from 8 to 25, with a mean of 15.8 (*SD* = 3.65) players. Participants came from 15 different nationalities, with a predominance of Norwegian participants (88.7%). Teams from the study sample were from seven different counties in Norway. Participants reported playing on their current team for a mean of 2.86 years (*SD* = 2.43), and 68 participants had represented their senior national team during the last three years.

### 2.2 Procedure

Clubs from the two highest senior levels in handball and ice hockey in Norway were contacted, either through their head coach or their sports director, about participating in the current study. Thirty-one clubs were asked, and 28 clubs agreed to participate. Data from these 28 teams was collected between November 2019 and March 2020. Three researchers gathered data individually via a hard copy questionnaire. Information about the study was given to the team verbally and in writing, prior to or after a training session, and it was made clear that participation was voluntary. Anonymity and confidentially were guaranteed to the participants, and they were also informed that they could withdraw from the study at any point. Participants used about 10–15 min to complete the questionnaire. Questionnaires completed by each player were gathered and placed in an envelope. Procedures were in line with the ethical standards of the first author's university and the Norwegian Social Sciences Data Service.

### 2.3 Measures

*Identity leadership* was measured using the Norwegian version [42] of the four-dimensional Identity Leadership Inventory (ILI) [31] comprising 15 items in total. Identity prototypicality was measured with four items (e.g., “My head coach is a model member of the team”), identity advancement with four items (e.g., “My head coach acts as a champion for the team”), identity entrepreneurship with four items (e.g., “My head coach creates a sense of cohesion within the team”) and identity impresarioship with three items (e.g., “My head coach creates structures that are useful for team members”). Two modifications were made to the ILI to make it more sports specific: “Leader” was substituted with “Head coach”, and “group” was substituted with “team”. Participants responded to items on a seven-point Likert scale (1 = *totally disagree*, 7 = *totally agree*). Higher scores reflected perceptions of stronger identity leadership. Identity leadership was estimated as a global second-order construct, with the four subdimensions at first-order level. This model has been shown to yield acceptable model-fit [31; model b, study 3].

*Intra-team communication* was measured using two subscales from the Scale of Effective Communication in Team Sports (SECTS-2) [38]: the dimension of acceptance (four items) and the dimension of distinctiveness (three items). For the purposes of this study, items were forward/backward translated to Norwegian according to recommendations by Kvamme et al. [43]. Items were rated on a seven-point Likert scale (1 = *Hardly ever*, 7 = *Almost always*). Higher scores reflected a greater amount of perceived intra-team communication.

*Task cohesion* was assessed using the Norwegian version of the Group Environmental Questionnaire [44]. The GEQ contains 18 items measuring task and social cohesion, but for the purposes of this study, only the task cohesion dimension was considered. It captures the beliefs the group and each member have regarding their team membership from a task perception and contains nine items. One item (“I am happy with the amount of playing time I get”) was deemed not relevant to the elite context in the current study [44,45] and was therefore removed without further analysis. Items were scaled with a nine-point Likert Scale (1 = *Never*, 9 = *Always*). Higher scores suggest greater perceived team task cohesiveness.

### 2.4 Statistical analyses

Within the framework of structural equation modeling (SEM), we tested the hypothesized model with the constructs of interest (identity leadership, intra-team communication, and task cohesion) as latent variables. The models were estimated with the full information maximum likelihood estimator (ML) using M*plus* v8.6 [46]. Item-level missing data were accounted for by the ML [47]. The chi-square test of exact fit is normally considered sensitive to sample size and minor model misspecifications [48]. Thus, model fit was evaluated with several goodness-of-fit indices and criteria: the Tucker Lewis index (TLI) > 0.90, comparative fit index (CFI) > 0.90, root mean square error of approximation (RMSEA) < 0.08 and the standardized root mean square residual (SRMR) < 0.08 [49]. To account for the nested data structure (clusters of teams), we adjusted the standard errors and goodness-of-fit model testing using Muthen and Satorra's [50] aggregated analysis (i.e., TYPE = COMPLEX in M*plus*).

The path analysis included one exogenous factor (second-order identity leadership with four first-order factors), two parallel mediators (acceptance and distinctiveness), and one endogenous factor (task cohesion). All direct, indirect, and total effects in the model were estimated with a bootstrapping procedure [51]. A bias-corrected bootstrapped 95% confidence interval that does not include zero is considered statistically significant.

Prior to estimating the structural model, the psychometric properties of the instruments were tested through separate measurement models. The measurement models were tested with the independent clusters model confirmatory factor analysis (ICM-CFA) approach given sufficient a priori measurement theory for these constructs. Each latent variable was measured with its respective observed indicators. Composite reliability was estimated with McDonald's [52] ω = (*Σ|λi|)*^*2*^*/([Σ|λi|*^*2*^*]* + *Σδii*) using standardized parameter estimates from the ICM-CFA models where *λi* are the factor loadings and *δii* are the error variances. McDonald's omega coefficient can be interpreted in a similar manner to the coefficient alpha, but it is a more flexible alternative for reliability estimation and does not rely on the tau-equivalence assumption [53].

### 2.5 Preliminary analyses

The result from the preliminary analyses indicated that the second-order identity leadership model yielded close-to acceptable fit indices (*S*–B χ2 = 444.882 [df = 86, N = 436], p < .001; CFI = 0.94; TLI = 0.93; RMSEA = . 098 [0.089–0.107], and SRMR = 0.050). Similarly, the two dimensions of acceptance and distinctiveness from SECTS-2 also yielded close-to-acceptable model-fit (*S*–B χ2 = 504.633 [df = 87, N = 436], p < .001; CFI = 0.93; TLI = 0.92; RMSEA = 0.105 [0.096–0.114], and SRMR = 0.052). Although the RMSEA values were marginally higher than a traditional threshold of 0.08, we decided to proceed with the models considering the controversy connected with post hoc modification of estimated models [54,55].^1^

The one-dimensional ICM-CFA of the task items from GEQ resulted in an acceptable model fit (*S*–B χ2 = 71.771 [df = 20, N = 438], p < .001; CFI = 0.98; TLI = 0.97; RMSEA = . 077 [0.058–0.096], and SRMR = 0.036).

## 3 Results

Inspection of skewness and kurtosis revealed that all the items generally fell within the cut-off values of ±2 [56]. All the items loaded as statistically significant on their respective latent constructs. Latent factor correlations are presented in Table 1. As can be seen, identity leadership had a strong positive statistically significant correlation to task cohesion. As shown in the Methods section, ω was estimated as an indicator of composite reliability, and based on .70 as cut-off [56], the ω values were acceptable for all the latent factors (range 0.70–0.93).

**Table 1:** Latent factors correlations matrix.

	2. Adv	3. Entr	4. Impr	5. Idl	6. Acc	7. Dist	8. Task
1. Prot	.904**	.817**	.690**	.925**	.515**	.119	.652**
2. Adv	–	.862**	.729**	.977**	.544**	.125	.689**
3. Entr		–	.659**	.883**	.492**	.113	.622**
4. Impr			–	.746**	.415**	.096*	.526**
5. Idl				–	.557**	.128	.705**
6. Acc					–	.312**	.808**
7. Dist						–	.173*

Note. *p < .05, **p < .01.

### 3.1 Testing the indirect effect

**Fig. 1:**
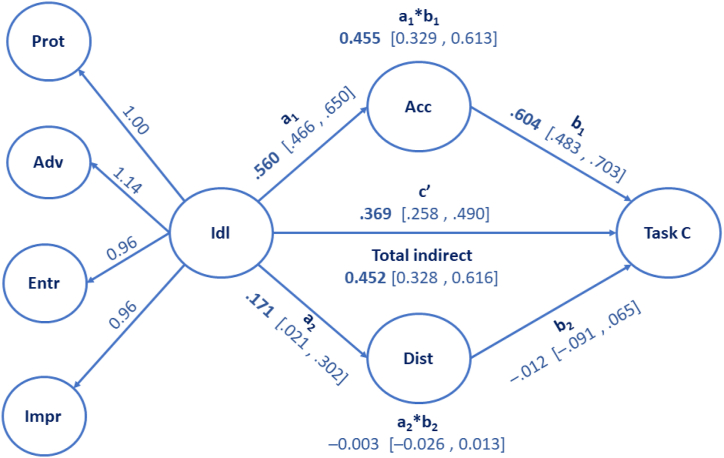
Visual presentation of results from the structural model, testing indirect effects of acceptance and distinctiveness in the relationship between identity leadership and task cohesion. Note. Paths are presented with standardized point estimates and 95% CI. Indirect estimations are presented as unstandardized bootstrapped estimates (with bias-corrected 95% CI). CI not including zero indicates statistically significant paths. Statistically significant point estimated in bold. N_replications_ = 10,000.

The structural model, where we controlled for cluster effects, yielded an acceptable model fit (*S*–B χ2 = 1001.232 [df = 396, N = 441], p < .001; CFI = 0.93; TLI = 0.93; RMSEA = 0.059 [0.054–0.063, and SRMR = 0.051). The bootstrapped estimates (with 95% bias-corrected CI) are shown in Fig. 1. As can be seen, there was a statistically significant positive relationship between identity leadership and task cohesion (c-path). Moreover, the proposed mediator acceptance significantly predicted task cohesion (b_1_-path), whereas the mediator distinctiveness did not (b_2_-path). Furthermore, identity leadership significantly and positively predicted the mediator acceptance (a_1_-path) and had a weak significant positive effect on distinctiveness (a_2_-path). Overall, there was a statistically significant indirect effect of identity leadership on task cohesion through acceptance (a_1_*b_1_-path).

## 4 Discussion

The purpose of this study was firstly to examine the relationship between identity leadership and task cohesion among elite team athletes, and secondly to explore whether intra-team communication (i.e., acceptance and distinctiveness) mediated this relationship. Using a sample of elite athletes from the two highest divisions in handball and ice hockey in Norway, the study aimed to address limitations within the existing literature on identity leadership in sport, including the lack of studies on elite athletes and the need to test potential mediators between identity leadership and key outcomes [19]. The results support the hypothesis of a positive relationship between identity leadership and task cohesion. Furthermore, the intra-team communication dimension acceptance was a significant mediator between identity leadership and task cohesion, whereas distinctiveness was not. As such, the results from the mediation analyses were partly in line with our a priori prediction.

The positive relationship between identity leadership and task cohesion lends support to the findings of Steffens et al. [31] who found positive associations between task cohesion and two of the four subdimensions of identity leadership (i.e., identity impresarioship and identity entrepreneurship). Previous studies have also demonstrated a link between social identity and cohesion [28] and between leadership styles aligning with the principles of social identity leadership and task cohesion (e.g., transformational leadership [10]). Perhaps more importantly, the positive relationship between identity leadership and task cohesion was expected, given the theoretical connections between identity leadership and task cohesion [31]. Indeed, the social identity approach to leadership is centered around leaders' capabilities to foster a shared social identity for group members [23,31], whereas task cohesion refers to a group's shared commitment to achieve common goals/objectives [4]. Identity leadership behaviors by coaches for elite sports groups can include engaging in activities beyond what is expected to increase the likelihood of reaching set goals and focusing on “we” and “us” rather than “I” and “me” [29]. By acting in accordance with identity leadership principles, coaches are likely to be perceived as acting for the group rather than for themselves, with a likely consequence of increased cohesiveness [26].

The present study identified a positive relationship between identity leadership and the mediating variable of intra-team communication acceptance. This relationship may be understood through shared identity in the team. Previous research has found a positive relationship between identity leadership and a shared sense of identity among followers [30,57]. Furthermore, shared identity has a distinct implication for the individual's cognition, emotion, and behavior [58,59]. For example, when people are identified with their group, they will be more willing to act cooperatively within the group and to invest their time and energy in working to see the group succeed. In the process of developing a shared identity, the leader's identity behavior is a key driver and provides a platform for psychological connection, communication, and sense of belonging [60]. This in turn may increase team members' willingness to share personal experiences, beliefs, values, attitudes, and personal motives. However, mutual sharing requires open and trustful communication (e.g., acceptance), which may explain the positive relationship between social identity leadership behavior and intra-team communication acceptance.

The positive relationship identified between the mediator acceptance and task cohesion in elite teams may also be understood in light of previous research and theory. According to the theory of cohesion, similarity in attitudes, beliefs, and motives may be considered as an antecedent to cohesion [8,61]. In elite sport, where the focus is primarily task and performance related [62,63], it is reasonable to believe that a relatively large part of the intra-team communication is related to task objectives including ambitions, goals, teamwork, and coordination. In this way, social identity in an elite context may promote shared agreement about goals, teamwork, and norms for contribution and effort, in addition to optimizing and strengthening them, and therefore may explain the relationship between intra-team communication acceptance and task cohesion.

Our results identified a significant positive relationship between identity leadership and intra-team communication distinctiveness. This result is in line with the theoretical reasoning that social identity creates contours and boundaries of communication [23] and draws parallel to the study by Smith et al. [34] who fund leadership behaviors to be related to intra-team communication. For example, when leaders promote social identity to a group, and the group becomes a relatively stable part of each team member's self-definition, the team more easily develops a shared and distinctive form of team communication [29,64]. Moreover, distinctiveness may be affected by the time team members play together on their current team and how long they work under the same coach [65]. Still, according to Bakar and Sheer [66], communication in teams can be divided into vertical (coach–athlete) and horizontal (athlete–athlete) communication. It is reasonable to believe that the unique team language among athletes is mainly initiated and developed in a horizontal pattern among athletes, based on insight and knowledge among team members, without the coach necessarily actively or explicitly contributing [67]. Considering the relatively weak association found in the present study, there may be a possibility that distinctiveness in communication within elite teams is primarily developed horizontally.

Previous research has suggested that distinctiveness is a positive predictor of cohesion [36,40]. Surprisingly, our results did not show a significant relation between distinctiveness and task cohesion. According to Ronglan [67], team language can be separated into two different categories: *on the pitch* and *off the pitch*, indicating different kind of relations and communication within the team. Therefore, participants' perception of distinctiveness may refer to off-pitch communications such as social and humoristic verbal and nonverbal team member interactions. This would support findings from McLaren and Spink [68] and Sullivan and Short [38] that distinctiveness is positively related to social cohesion. Nevertheless, distinctiveness measured in this study might primarily be perceived as relating to off-pitch communication (e.g., social and humoristic) and therefore we argue that distinctiveness would to a lesser degree be related to task cohesion, and rather probably be more related to social cohesion. Even when distinctiveness is developed through horizontal communication, the coach's ability to facilitate positive communication patterns through identity leadership would still be an important factor, and therefore may contribute without being the primary driver of team communication processes on and off the pitch [69]. While this may indicate that elite teams have distinctive verbal and nonverbal communication patterns on and off the pitch, such differences were not investigated in the present study.

The results from the present study are not without their limitations, and these should be considered when interpreting our findings. First, even though measures used in this study have been validated and used in previous research [31,38,44], investigating elite athletes can be considered an extreme case; thus, it is unclear whether measures used in this study are sufficiently specific in an elite-sports context. In addition, it must be considered that our results depend on athletes' subjective ratings, which can be influenced by selective memory or halo effects [19]. Factors such as date and time, stress or flow, and win–loss record, may have influenced players’ ratings when they filled out our questionnaires. Second, only two different team sports (ice hockey and handball) are represented in our study sample. However, with limited research investigating identity leadership and elite athletes, we consider it a strength that our sample size comprises 441 elite athletes representing both male and female athletes from the two highest divisions in Norway in two different sports. Lastly, causation and longitudinal trends caused by identity leadership cannot be investigated with our cross-sectional design.

Future studies would benefit from an in-depth longitudinal exploration of elite coaches' use of identity leadership in a naturalistic setting similar to that used by Herbison et al. [21] to explore both positive and negative effects of the four dimensions of identity leadership. This could give an insight into elite coaches’ actual identity leadership behaviors, and their relation to important group processes and outcomes. Moreover, knowledge about how elite coaches integrate identity leadership into their day-to-day practice with athletes should be expanded. In addition, it would be fruitful to investigate how personal (e.g., “I” and “me”) and collective (e.g., “we” and “us”) language is used in structured and unstructured team activities, and how this influences individual and team outcomes (e.g., cohesion and collective efficacy) among elite athletes. This could help explain how different verbal and nonverbal communication on and off the pitch develops, and to what extent it is auto-generated among team members. Also, exploring and further developing the dimension of distinctiveness and the possibility of separately measuring the social and task-related contents of distinctiveness would be of great interest. Lastly, future research could benefit from investigating the unique contribution made by each dimension of identity leadership related to cohesion and intra-team communication.

In summary, the current study adds insight into identity leadership in an elite team context. Our findings suggest a positive relationship between identity leadership and task cohesion, with acceptance as a significant mediator between identity leadership and task cohesion. As noted by Stevens et al. [19], little research has been conducted on identity leadership in elite sports, and this study therefore expands our understanding of the performance-related benefits of identity leadership.

## Declaration of competing interest

The authors declare that they have no known competing financial interests or personal relationships that could have appeared to influence the work reported in this paper.

## Footnotes

1 Similar to Steffens et al. [31], a one-factor first-order model of identity leadership yielded nonacceptable model fit (*S*–B χ2 = 1113.363 [df = 90, N = 436], p < .001; CFI = 0.83; TLI = 0.80; RMSEA = 0.161 [0.153–0.170], and SRMR = 0.069).
