# Supplementary material for: Identity leadership and cohesion in elite sport: The mediating role of intra-team communication
Source: Heliyon. 2023 Jul 3;9(7):e17853. doi: 10.1016/j.heliyon.2023.e17853 (PMC10345360; doi:10.1016/j.heliyon.2023.e17853)
Supplement: Multimedia component 1 [file mmc1.docx]

**
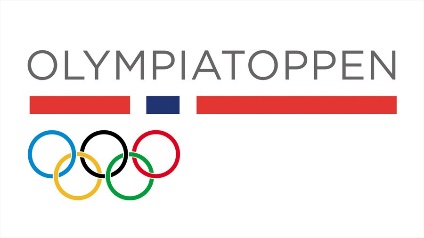
**

**
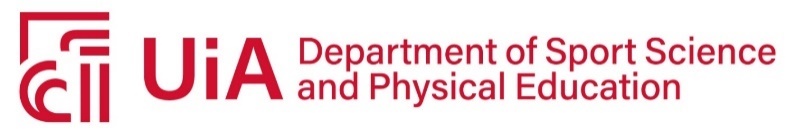
**

| Elite Team Prosjekt 2022 |
| --- |

**Kjære deltaker!**

Denne undersøkelsen er en del av Elite Team Project 2022 initiert av Universitetet i Agder i samabeid med Olympiatoppen Sør, der målet er utvikle ny og nyansert kunnskap om psykologiske og sosiale faktorer i eliteteam.

Først og fremst håper vi at du vil delta i denne undersøkelsen og tar deg tid til å svare på spørsmålene.

Alle svarene vil bli behandlet konfidensielt. Du og ditt lag vil ikke kunne bli identifisert i den senere rapporteringen av resultatene i det vitenskapelige arbeid. Prosjektet er meldt inn og godkjent av NSD og Fakultetets etikkkomite ved universitetet i Agder.

| **Rune Høigaard**  Professor  Universitetet i Agder  Fakultet for helse og idrett  Serviceboks 422  4604 Kristiansand  Tlf (j) 38141253  e-post: rune.hoigaard@uia.no | **Gaute Schei**  Doktorgradsstipendiat  Universitetet i Agder  Fakultet for helse og idrett  Serviceboks 422  5602 Kristiansand  Tlf 38141579  e-post: gaute.s.schei@uia.no | **Tommy Haugen**  Førsteamanuensis  Universitetet i Agder  Fakultet for helse og idrett  Serviceboks 422  5602 Kristiansand  Tlf 38142327  e-post: tommy.haugen@uia.no | |  |
| --- | --- | --- | --- | --- |
|  |  |  |  | |

| **Informasjon**  De fleste spørsmålene i spørreskjemaet er basert på at du skal vurdere ulike beskrivelser, påstander og utsagn på en skala (for eksempel fra helt enig til helt uenig). *Sett en ring rundt det tallet som passer best for deg.* Det finnes ingen rette eller gale svar, det er din oppfatning som er av interesse.  Legg merke til at skalaene kan variere noe fra spørsmål til spørsmål, så det er viktig at du er oppmerksom på det. Selv om enkelte spørsmål kan oppleves som like er det viktig at du svarer på alle spørsmålene.  Lag og gruppe blir brukt litt omhverandre men er det samme, dvs den idrettgruppen du tilhører og som du trener, spiller eller konkurrerer sammen med. Spillere, utøver og medlemmer blir også brukt om hverandre, men betyr det samme, dvs. de som er knyttet til din gruppe/lag. Dersom du har spørsmål ved studien kan du ta kontakt med Gaute Schei, Tommy Haugen eller Rune Høigaard. |
| --- |

**Litt om deg selv**

**Alder:**____________

**Nasjonalitet:**____________

**Hvor lenge har du spilt på dette laget?**_______ år

**Hvor mange år har du spilt i eliteserien eller 1 divisjon (totalt) ?**_______ år

**Har du spilt (vært uttatt) til et landslag på seniornivå de siste 3 årene?** Nei Ja

**Har du spilt for en utenlandsk klubb i øverste divisjon eller 1 divisjon på seniornivå?** Nei Ja _______ år

**Mitt team/lag**

I denne delen skal du vurdere humor, kommunikasjon og samhold i ditt team/ lag.

| **I mitt lag..** | Helt uenig | |  | | | | | | Helt  enig |
| --- | --- | --- | --- | --- | --- | --- | --- | --- | --- |
| Finner spillere på humoristiske påfunn | 1 | 2 | | 3 | 4 | 5 | 6 | 7 | |
| Tuller spillere med hverandre  (vitser, imitasjon, kommentarer, narrestreker) | 1 | 2 | | 3 | 4 | 5 | 6 | 7 | |
| Forteller spillere morsomme vitser som skaper smil og lattter | 1 | 2 | | 3 | 4 | 5 | 6 | 7 | |
| Opplever jeg vennlig ironi | 1 | 2 | | 3 | 4 | 5 | 6 | 7 | |
| Forteller spillere negative historier om hverandre for å være morsom | 1 | 2 | | 3 | 4 | 5 | 6 | 7 | |
| Er humoren slik at enkelte føler seg mindre | 1 | 2 | | 3 | 4 | 5 | 6 | 7 | |
| Er humoren preget av diskriminerende innhold | 1 | 2 | | 3 | 4 | 5 | 6 | 7 | |
| Bruker spillere og trenere negativ humor om hverandre for å være morsom | 1 | 2 | | 3 | 4 | 5 | 6 | 7 | |
| Blir humor om medspillere brukt på en krenkende måte | 1 | 2 | | 3 | 4 | 5 | 6 | 7 | |
| Imiteres personer utenfor laget på en respektløs måte  (støtteapparat, spillere på andre lag, dommere, supportere, journalister etc.) | 1 | 2 | | 3 | 4 | 5 | 6 | 7 | |
| Brukes ondsinnet humor om personer i idrettsmiljøet  (støtteapparat, spillere på andre lag, dommere, supportere, journalister etc.) | 1 | 2 | | 3 | 4 | 5 | 6 | 7 | |
| Bruker spillere støtende humor om personer utenfor laget  (støtteapparat, spillere på andre lag, dommere, supportere, journalister etc.) | 1 | 2 | | 3 | 4 | 5 | 6 | 7 | |
| Ler spillere av diskriminerende kommentarer om personer utenfor laget (støtteapparat, spillere på andre lag, dommere, supportere, journalister etc.) | 1 | 2 | | 3 | 4 | 5 | 6 | 7 | |
| Bruker spillere fiendtlig humor om personer utenfor laget  (støtteapparat, spillere på andre lag, dommere, supportere, journalister etc.) | 1 | 2 | | 3 | 4 | 5 | 6 | 7 | |

| **Når vi i vårt lag kommuniserer…** | Nesten  aldri | |  | | | | | Nesten  alltid | |
| --- | --- | --- | --- | --- | --- | --- | --- | --- | --- |
| Bruker vi kallenavn | 1 | 2 | | 3 | 4 | 5 | 6 | | 7 |
| Stoler vi på hverandre | 1 | 2 | | 3 | 4 | 5 | 6 | | 7 |
| Uttrykker vi følelsene våre åpent og ærlig | 1 | 2 | | 3 | 4 | 5 | 6 | | 7 |
| Bruker vi internt språk som bare lagets medlemmer forstår | 1 | 2 | | 3 | 4 | 5 | 6 | | 7 |
| Bruker vi fakter og tegn som bare lagets medlemmer forstår | 1 | 2 | | 3 | 4 | 5 | 6 | | 7 |
| Deler vi tanker med hverandre | 1 | 2 | | 3 | 4 | 5 | 6 | | 7 |
| Prøver vi å sørge for at alle er inkludert | 1 | 2 | | 3 | 4 | 5 | 6 | | 7 |

| **Meg og min gruppe/lag** | | Svært  uenig | | | | | Svært  enig | | | | | |
| --- | --- | --- | --- | --- | --- | --- | --- | --- | --- | --- | --- | --- |
|  | |  | |  |  |  |  | |  |  |  |  |
| Jeg er fornøyd med den grad av interesse laget mitt har for å vinne | | 1 | | 2 | 3 | 4 | 5 | | 6 | 7 | 8 | 9 |
| Dette laget gir meg tilstrekkelige muligheter til å utvikle meg til en bedre spiller/utøver | | 1 | | 2 | 3 | 4 | 5 | | 6 | 7 | 8 | 9 |
| Jeg liker måten dette laget utøver idretten sin på (f.eks. spillestilen) | | 1 | | 2 | 3 | 4 | 5 | | 6 | 7 | 8 | 9 |
| Laget vårt står sammen i forsøket på å oppnå de målene vi har satt oss | | 1 | | 2 | 3 | 4 | 5 | | 6 | 7 | 8 | 9 |
| Vi tar alle ansvar dersom laget ”taper” eller oppnår dårlige resultater | | 1 | | 2 | 3 | 4 | 5 | | 6 | 7 | 8 | 9 |
| Spillerne på laget vårt har like ambisjoner når det gjelder lagets resultater | | 1 | | 2 | 3 | 4 | 5 | | 6 | 7 | 8 | 9 |
| Dersom noen av spillerne på laget vårt har problemer med en øvelse på trening, ønsker alle å hjelpe | | 1 | | 2 | 3 | 4 | 5 | | 6 | 7 | 8 | 9 |
| Spillerne på laget vårt snakker åpent om den enkelte spillers ansvar under kamp eller trening | | 1 | | 2 | 3 | 4 | 5 | | 6 | 7 | 8 | 9 |

**Min trener**

I denne delen skal du vurdere din trener

| **Min hovedtrener…** | Ikke i det  hele tatt  (Aldri) | En sjelden gang | Av og til | Ganske ofte | Svært ofte (Alltid) |
| --- | --- | --- | --- | --- | --- |
| Revurderer kritiske antagelser for å se om de er hensiktsmessige.  («Er det lurt å gjøre det på denne måten») | 1 | 2 | 3 | 4 | 5 |
| Snakker om sine viktigste verdier og overbevisninger | 1 | 2 | 3 | 4 | 5 |
| Prøver å få fram flere ulike perspektiver når problemer skal løses | 1 | 2 | 3 | 4 | 5 |
| Snakker optimistisk om fremtiden | 1 | 2 | 3 | 4 | 5 |
| Snakker entusiastisk om hva som må oppnås | 1 | 2 | 3 | 4 | 5 |
| Understreker betydningen av å være målbevisst | 1 | 2 | 3 | 4 | 5 |
| Bruker tid på opplæring og veiledning | 1 | 2 | 3 | 4 | 5 |
| Behandler spillere som individer/enkeltpersoner og ikke bare som medlemmer av en gruppe | 1 | 2 | 3 | 4 | 5 |
| Overveier moralske og etiske konsekvenser av beslutninger | 1 | 2 | 3 | 4 | 5 |
| Uttrykker utfordrende fremtidsvisjoner | 1 | 2 | 3 | 4 | 5 |
| Tar hensyn til at spillere har ulike behov, evner og ambisjoner | 1 | 2 | 3 | 4 | 5 |
| Får andre til å se problemer fra mange forskjellige synsvinkler | 1 | 2 | 3 | 4 | 5 |
| Hjelper spillerne til å utvikle sine sterke sider | 1 | 2 | 3 | 4 | 5 |
| Foreslår nye måter å løse oppgaver på | 1 | 2 | 3 | 4 | 5 |
| Legger vekt på betydningen av å ha en felles målsetning | 1 | 2 | 3 | 4 | 5 |
| Uttrykker tillit til at målsetninger blir nådd | 1 | 2 | 3 | 4 | 5 |

| **Min hovedtrener…** | Helt  uenig | |  | | | | | | Helt enig |
| --- | --- | --- | --- | --- | --- | --- | --- | --- | --- |
| Representerer det laget står for | 1 | 2 | | 3 | 4 | 5 | 6 | 7 | |
| Er representativ for laget | 1 | 2 | | 3 | 4 | 5 | 6 | 7 | |
| Er modell for laget | 1 | 2 | | 3 | 4 | 5 | 6 | 7 | |
| Er et foregangseksempel som gruppemedlem | 1 | 2 | | 3 | 4 | 5 | 6 | 7 | |
| Fremmer spillernes interesser | 1 | 2 | | 3 | 4 | 5 | 6 | 7 | |
| Forfekter lagets interesser | 1 | 2 | | 3 | 4 | 5 | 6 | 7 | |
| Taler lagets sak | 1 | 2 | | 3 | 4 | 5 | 6 | 7 | |
| Har alltid laget sine interesser i tankene | 1 | 2 | | 3 | 4 | 5 | 6 | 7 | |
| Gir spillerne følelsen av å tilhøre samme gruppe | 1 | 2 | | 3 | 4 | 5 | 6 | 7 | |
| Skaper følelsen av samhold i laget | 1 | 2 | | 3 | 4 | 5 | 6 | 7 | |
| Utvikler forståelse for gruppemedlemskap | 1 | 2 | | 3 | 4 | 5 | 6 | 7 | |
| Utvikler lagets forståelse for felles verdier og idealer | 1 | 2 | | 3 | 4 | 5 | 6 | 7 | |
| Tenker ut aktiviteter som samler gruppemedlemmene | 1 | 2 | | 3 | 4 | 5 | 6 | 7 | |
| Gjennomfører tiltak og “events” slik at laget fungerer effektivt | 1 | 2 | | 3 | 4 | 5 | 6 | 7 | |
| Utvikler nyttige gruppestrukturer | 1 | 2 | | 3 | 4 | 5 | 6 | 7 | |

| **Min hovedtrener…** | | Helt uenig | |  | | | | | | Helt  enig |
| --- | --- | --- | --- | --- | --- | --- | --- | --- | --- | --- |
| Gjør narr av meg | 1 | | 2 | | 3 | 4 | 5 | 6 | 7 | |
| Uttrykker at mine tanker og følelser er dumme | 1 | | 2 | | 3 | 4 | 5 | 6 | 7 | |
| Rakker ned på meg foran andre | 1 | | 2 | | 3 | 4 | 5 | 6 | 7 | |
| Kommer med negative kommentarer om meg til andre | 1 | | 2 | | 3 | 4 | 5 | 6 | 7 | |
| Forteller meg at jeg er dårlig/udugelig | 1 | | 2 | | 3 | 4 | 5 | 6 | 7 | |

**Meg som idrettsutøver**

I denne delen er vi interessert i din vurdering av deg selv som idrettsutøver.

| **Hvor ofte opplever du?** | | Nesten  aldri | | Sjeldent | | Iblant | | Ofte | | Nesten  alltid |
| --- | --- | --- | --- | --- | --- | --- | --- | --- | --- | --- |
| Jeg føler meg fornøyd med det jeg presterer (oppnår) i min idrett | 1 | | 2 | | 3 | | 4 | | 5 | |
| Jeg kjenner meg så sliten av all min trening, at jeg har vansker med å orke andre ting | 1 | | 2 | | 3 | | 4 | | 5 | |
| Den innsats som jeg har lagt ned i min idrett ville/kunne ha gjort mer nytte på andre områder | 1 | | 2 | | 3 | | 4 | | 5 | |
| Jeg kjenner meg altfor sliten på grunn av min idrettsutøvelse | 1 | | 2 | | 3 | | 4 | | 5 | |
| Jeg presterer ikke særlig bra i min idrett | 1 | | 2 | | 3 | | 4 | | 5 | |
| Jeg bryr meg ikke like mye om min idrettsprestasjon som jeg pleide tidligere | 1 | | 2 | | 3 | | 4 | | 5 | |
| Jeg presterer ikke opp til mitt fulle potensial (kapasitet) i min idrett | 1 | | 2 | | 3 | | 4 | | 5 | |
| Jeg føler meg utmattet på grunn av deltakelse i min idrett | 1 | | 2 | | 3 | | 4 | | 5 | |
| Jeg er ikke like interessert i min idrettsatsning som jeg pleide å være | 1 | | 2 | | 3 | | 4 | | 5 | |
| Jeg kjenner meg fysisk utslitt på grunn av min idrettsdeltakelse | 1 | | 2 | | 3 | | 4 | | 5 | |
| Jeg er mindre opptatt av å være fremgangsrik i min idrett, sammenlignet med sånn jeg pleide å være | 1 | | 2 | | 3 | | 4 | | 5 | |
| Jeg er utmattet av de mentale og fysiske belastningene i min idrett | 1 | | 2 | | 3 | | 4 | | 5 | |
| Det virker som uansett hva jeg gjør, så presterer jeg ikke like bra som jeg burde | 1 | | 2 | | 3 | | 4 | | 5 | |
| Jeg føler meg fremgangsrik i min idrett | 1 | | 2 | | 3 | | 4 | | 5 | |
| Jeg har negative følelser i forbindelse med min deltakelse i idrett | 1 | | 2 | | 3 | | 4 | | 5 | |

**Snu arket for å besvare de siste spørsmålene.**

| **Når jeg..** | | Helt uenig |  | | | | | Helt  enig |
| --- | --- | --- | --- | --- | --- | --- | --- | --- |
| Mislykkes begynner jeg å tvile på talentet mitt | 1 | | | 2 | 3 | 4 | 5 | |
| Mislykkes ødelegger det mine fremtidsplaner | 1 | | | 2 | 3 | 4 | 5 | |
| Ikke lykkes blir folk mindre interessert i meg | 1 | | | 2 | 3 | 4 | 5 | |
| Mislykkes blir personer som er viktige for meg skuffet | 1 | | | 2 | 3 | 4 | 5 | |
| Mislykkes blir jeg bekymret for hva andre tenker om meg | 1 | | | 2 | 3 | 4 | 5 | |

| **Jeg er fornøyd med..** | Ikke  fornøyd | | | Moderat  fornøyd | | | | | Ekstremt  fornøyd | |
| --- | --- | --- | --- | --- | --- | --- | --- | --- | --- | --- |
| I hvilken grad jeg gjør mitt beste for laget | | 1 | 2 | | 3 | 4 | 5 | 6 | | 7 |
| Min innsats og innstilling på trening | | 1 | 2 | | 3 | 4 | 5 | 6 | | 7 |
| Min entusiasme i kamp | | 1 | 2 | | 3 | 4 | 5 | 6 | | 7 |
| Min forpliktelse til laget | | 1 | 2 | | 3 | 4 | 5 | 6 | | 7 |

| **Tilfredshet med min rolle** | Helt  uenig | | |  | | | | | | | Helt  enig | |
| --- | --- | --- | --- | --- | --- | --- | --- | --- | --- | --- | --- | --- |
| Jeg er fornøyd med ansvaret og oppgavene jeg har fått tildelt i min rolle | | 1 | 2 | | 3 | 4 | 5 | 6 | 7 | 8 | | 9 |
| Jeg er fornøyd med den rollen jeg har fått tildelt | | 1 | 2 | | 3 | 4 | 5 | 6 | 7 | 8 | | 9 |
| Jeg liker å utføre oppgavene i min rolle | | 1 | 2 | | 3 | 4 | 5 | 6 | 7 | 8 | | 9 |
| Jeg liker ikke min rolle | | 1 | 2 | | 3 | 4 | 5 | 6 | 7 | 8 | | 9 |

**Tusen takk for at du har tatt deg tid til å besvare undersøkelsen**
