# Supplementary material for: Identity leadership and cohesion in elite sport: The mediating role of intra-team communication
Source: Heliyon. 2023 Jul 3;9(7):e17853. doi: 10.1016/j.heliyon.2023.e17853 (PMC10345360; doi:10.1016/j.heliyon.2023.e17853)
Supplement: Multimedia component 2 [file mmc2.docx]

The following studies are chronological references to included instruments in the attached questionnaire:

Humor climate: Schei et al. (2021). *Development and Initial Validation of the Humor Climate in Sport Scale.*

Intra-team communication: Sullivan & Short (2011). *Further operationalization of Intra-Team Communication in Sports: An Updated Version of the Scale of Effective Communication in Team Sports (SECTS-2).*

Cohesion: Haugen et al. (2021). *Psychometric Evaluation of the Norwegian Versions of the Modified Group Environment Questionnaire and the Youth Sport Environment Questionnaire.*

Transformational leadership: Bass & Avolio (1995). *Multifactor Leadership Questionnaire*.

Identity leadership: van Dick et al. (2018). *Identity leadership going global: Validation of the Identity Leadership Inventory across 20 countries.*

Destructive leadership: Mitchell & Ambrose (2007). *Abusive Supervision and Workplace Deviance and the moderating Effects of Negative Reciprocity Beliefs.*

Burnout: Raedeke & Smith (2001). *Development and Preliminary Validation of an Athlete Burnout measure.*

Fear of failure: Conroy et al. (2002). *Multidimensional Fear of Failure Measurement: The performance Failure Appraisal Inventory.*

Dedication: Riemer & Chelladurai (1998). *Development of the Athlete Satisfaction Questionnaire (ASQ).*

Role satisfaction: Beauchamp et al. (2005). *Multidimensional Role Ambiguity and Role Satisfaction: A prospective Examination Using Interdependent Sport Teams.*
